# Epidemiological characteristics of malaria from control to elimination in Hubei Province, China, 2005–2016

**DOI:** 10.1186/s12936-018-2207-7

**Published:** 2018-02-15

**Authors:** Jing Xia, Xibao Huang, Lingcong Sun, Hong Zhu, Wen Lin, Xiaorong Dong, Dongni Wu, Juan Qiu, Li Zheng, Mumin Cao, Si Liu, Huaxun Zhang

**Affiliations:** 1 Institute of Parasitic Disease Control, Hubei Provincial Center for Disease Control and Prevention, Wuhan, 430079 China; 2 Key Laboratory of Monitoring and Estimate for Environment and Disaster of Hubei Province, Institute of Geodesy and Geophysics, Chinese Academy of Sciences, Wuhan, 430077 China

**Keywords:** Malaria, Epidemiology, Imported cases, Spatiotemporal analysis, Elimination, Hubei Province, China

## Abstract

**Background:**

Hubei Province, China, has been operating a malaria elimination programme. This study aimed at investigating the epidemiologic characteristics of malaria in Hubei Province (2005–2016) to plan resource allocation for malaria elimination.

**Methods:**

Data on all malaria cases from 2005 to 2016 in all counties of Hubei Province were extracted from a web-based reporting system. The numbers of indigenous and imported cases during the disease control (2005–2010) and elimination (2011–2016) stages, as well as their spatiotemporal distribution, were compared.

**Results:**

A total of 8109 malaria cases were reported from 2005 to 2016 (7270 and 839 cases during the control and elimination stages, respectively). Between 2005 and 2010, indigenous malaria cases comprised the majority of total cases (7114/7270; 97.9%), and *Plasmodium vivax* malaria cases accounted for most malaria cases (5572/7270; 76.6%). No indigenous malaria cases have been reported in Hubei Province since 2013. Imported malaria cases showed a gradually increasing trend from 2011 to 2016, *Plasmodium falciparum* was the predominant species in these cases, and the number of counties with imported cases increased from 4 in 2005 to 47 in 2016. During the control and elimination stages, the most likely spatial clusters for indigenous cases included 13 and 11 counties, respectively. However, the cluster of indigenous malaria cases has not been identified since September 2011. For imported cases, the most likely cluster and three secondary clusters during both stages were identified.

**Conclusions:**

Hubei Province has made significant achievements in controlling and eliminating malaria; however, the region now faces some challenges associated with the increasing number and distribution of imported malaria cases. Priorities for malaria elimination should include better management of imported malaria cases, prevention of secondary malaria transmission, and ensuring the sustainability of malaria surveillance.

## Background

Malaria remains a major public health issue. According to the latest global estimates, there were 212 million new cases of malaria and 429,000 deaths attributable to the disease in 2015 [1]. Malaria is a parasitic disease that has been common throughout the history of China [2]. Intensive efforts at controlling the disease have been made over the past decades, and malaria cases in China have decreased from 61,204 cases  in 2006 to 14,278 cases in 2009 [3]. To protect public health and to achieve the global goal of malaria eradication, the Chinese Government developed a National Malaria Elimination Programme (NMEP) in 2010. China’s goal was to eliminate indigenous malaria by 2015 in the majority of the country’s regions with the exception of the Yunnan–Myanmar border areas and to completely eliminate malaria across China by 2020 [4]. In accordance with this goal, strategies and measures need to be adjusted from control to elimination. The aim of the control stage was to reduce the morbidity and mortality of malaria among the population [5]. However, the malaria elimination stage requires the detection and response to individual malaria cases, and elimination of the source of infection to ultimately prevent local malaria transmission [6, 7]. Since 2010, substantial progress in malaria elimination has been made in China [3, 8]. The indigenous malaria burden in China has decreased, and locally transmitted cases decreased from 1469 cases in 2011 to 43 cases in 2015 [9].

Historically, malaria transmission has been unstable and prone to outbreaks in Hubei Province. Malaria has been prevalent in this region, and widespread outbreaks occurred in the 1950 and 1970s [10]. After decades of control efforts, the incidence of malaria reduced significantly, from 3688.9/100,000 population in 1973 to 1.2/100,000 population in 2009 [11]. As the NMEP was launched in May 2010, Hubei Province has undergone the change from the disease control (2005–2010) to elimination stage (2011–2016). While studies on the changes in epidemiological malaria characteristics between disease control and elimination stages have been conducted in China [3, 5, 12, 13], few of these studies investigated the changes in malaria epidemic characteristics from the control stage (2005–2010) to the elimination stage (2011–2016) in Hubei Province. In this study, the epidemiological characteristics of malaria in Hubei from 2005 to 2016 were described and analysed, so as to help devise a strategy for resource allocation for malaria elimination in Hubei Province.

## Methods

### Study area

**Fig. 1:**
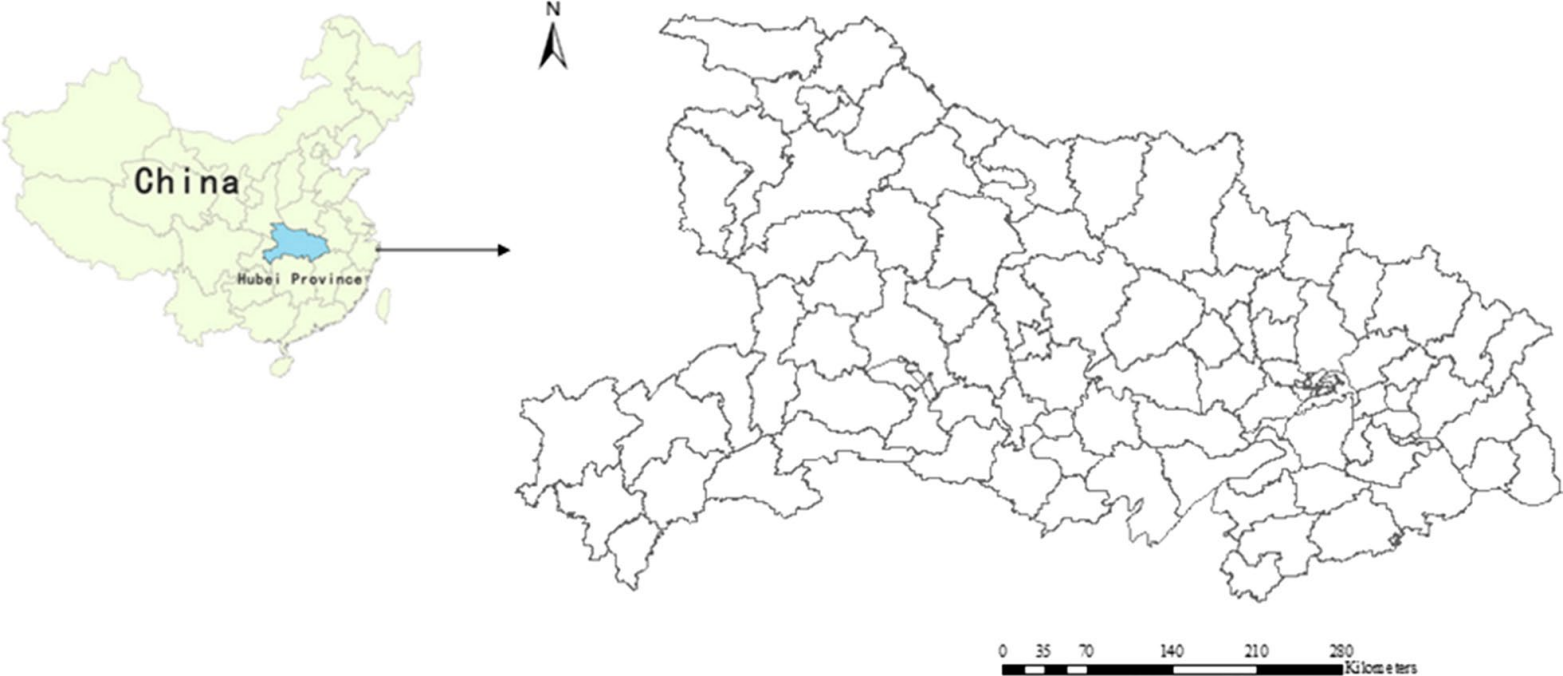
Location of Hubei Province, China

Hubei Province is located on the middle and lower reaches of the Yangtze River, which lies on the south-central mainland of China. The province has a population of 57.2 million and covers 185,900 sq km. It includes 102 counties, and Wuhan City is the provincial capital (Fig. 1).

### Data collection

This retrospective study was conducted to compare the characteristics of malaria epidemiology between disease control and elimination stages. Data on malaria cases at county level in Hubei Province from 2005 to 2016 were obtained from the China Information System for Disease Control and Prevention (CISDCP). All clinically diagnosed cases and laboratory-confirmed cases were reported to the CISDCP. Clinically diagnosed cases were defined as individuals presenting with malaria-like symptoms, with a history of travel to a malaria-endemic area during the malaria transmission season, but showing no signs of malaria with a laboratory test. Laboratory-confirmed cases were defined as individuals with confirmed disease through microscopic examination, rapid diagnostic testing (RDT), or polymerase chain reaction (PCR). Both types of cases were included in this study.

In China, an indigenous malaria case is defined as a malaria infection that was acquired locally and in which the patient does not have a history of travel. An imported case is defined as an individual having a history of travel to a malaria-endemic country outside China and an onset of malaria of < 1 month after returning to China from a malaria-endemic country.

In this study, the period from 2005 to 2010 was defined as the control stage and the period from 2011 to 2016 as the elimination stage.

### Seasonal fluctuation analysis

A seasonal index for each month from 2005 to 2016 was used to identify the seasonal patterns of malaria incidence (including both indigenous and imported cases). The index was calculated as the average of the number of cases in a given month (e.g., July 2005) divided by the mean of number of cases in the corresponding month during the 12 years of analysis (e.g., July 2005–2016). There was no seasonal fluctuation when the seasonal index for a given month was close to 1.0.

### Space–time clusters analysis

A space–time scan statistic was employed to test whether malaria cases were distributed randomly over space and time, and if they were not, to detect space–time clusters and evaluate their statistical significance. SaTScan software (version 9.4, Kulldorff and Information Management Services, Inc.) was used to conduct this retrospective space–time scan statistic [14, 15]. The maximum spatial cluster size of the population at risk was set to 50%, and the maximum temporal cluster size was 50% within the study period. The most significant clusters as well as secondary clusters were identified. The ArcGIS software version 10.0 (ESRI Inc., Redlands, CA, USA) was used to visualize space–time clusters of indigenous and imported malaria cases.

## Results

### General epidemiological malaria characteristics during the control and elimination stages

A total of 8109 malaria cases were reported in Hubei Province from 2005 to 2016. The number of reported cases reached a peak in 2007 (N = 1769). At total of 7270 and 839 cases were identified during the control (2005–2010) and elimination (2011–2016) stages, respectively. Indigenous malaria cases accounted for the majority of total cases during the control stage (7114/7270; 97.9%). Overall, the number of indigenous malaria cases dropped sharply between 2005 and 2010, decreasing significantly from 1513 in 2005 to 390 in 2010. No indigenous malaria cases have been reported in Hubei Province since 2013. The number of imported malaria cases showed a gradually increasing trend from 2011 to 2016. During the elimination stage, imported cases accounted for 89.4% (750/839) of all malaria cases. Moreover, the number of counties with imported cases increased from 4 in 2005 to 47 in 2016 (Table 1).

**Table 1:** Descriptive statistics of malaria cases during the control and elimination stages in Hubei Province

Characteristics	Control stage (2005–2010)						Elimination stage (2011–2016)					
	2005	2006	2007	2008	2009	2010	2011	2012	2013	2014	2015	2016
Total cases	1518	1753	1769	1089	712	429	167	132	129	140	120	151
Indigenous case (% of total cases)	1513 (99.7)	1746 (99.6)	1720 (97.2)	1064 (97.7)	681 (95.6)	390 (90.9)	80 (47.9)	9 (6.8)	0 (0)	0 (0)	0 (0)	0 (0)
Imported case (% of total cases)	5 (0.3)	7 (0.4)	49 (2.8)	25 (2.3)	31 (4.4)	39 (9.1)	87 (52.1)	123 (93.2)	129 (100.0)	140 (100.0)	120 (100.0)	151 (100.0)
No. county with cases (% of total counties)	66 (64.7)	65 (63.7)	69 (67.6)	63 (61.8)	60 (58.8)	60 (58.8)	52 (51.0)	38 (37.3)	36 (35.3)	45 (44.1)	40 (39.2)	47 (46.1)
No. county with indigenous cases (% of total counties)	66 (64.7)	63 (61.8)	65 (63.7)	55 (53.9)	53 (52.0)	52 (51.0)	19 (18.6)	4 (3.9)	0 (0)	0 (0)	0 (0)	0 (0)
No. county with imported cases (% of total counties)	4 (3.9)	6 (5.9)	16 (15.7)	14 (13.7)	19 (18.6)	23 (22.5)	42 (41.2)	37 (36.3)	36 (35.3)	45 (44.1)	40 (39.2)	47 (46.1)

**Fig. 2:**
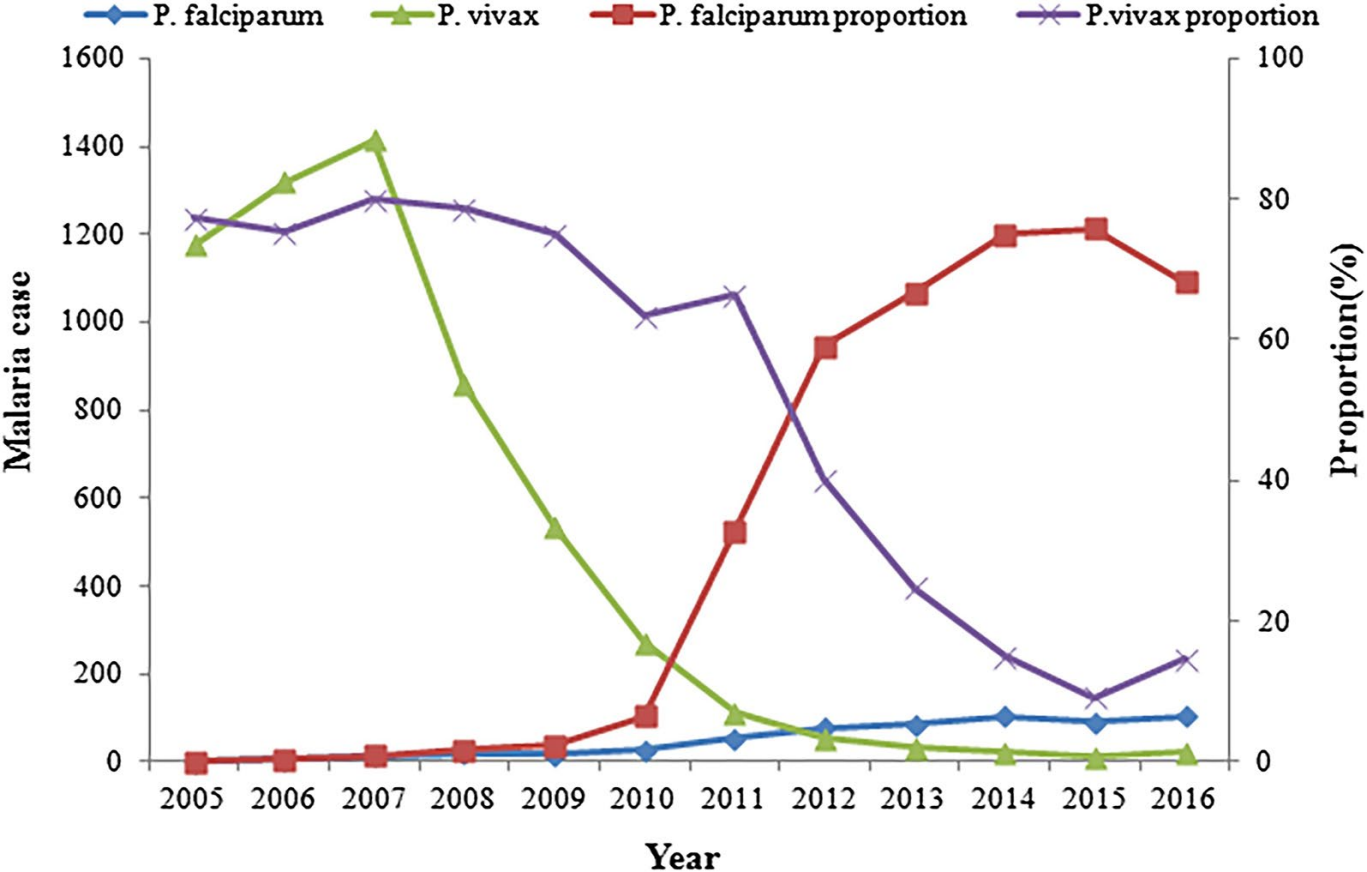
Proportion of *Plasmodium vivax* and *Plasmodium falciparum* in total cases in Hubei Province, 2005–2016

*Plasmodium vivax* malaria cases accounted for the majority of total malaria cases during the control stage (5572/7270; 76.6%). In contrast, most malaria cases were attributed to *Plasmodium falciparum* infection (518/839; 61.7%) during the elimination stage. The proportion of malaria cases associated with *P. falciparum* increased from 0.1% in 2005 to 68.2% in 2016 (Fig. 2).

The majority of malaria cases (80.7%; 5865/7270) were clinically diagnosed cases in the control stage. During the elimination stage, clinically diagnosed cases accounted for 4.1% (34/839) of total cases. All malaria cases were laboratory-confirmed cases since 2012.

### Season fluctuation of indigenous and imported malaria cases

**Fig. 3:**
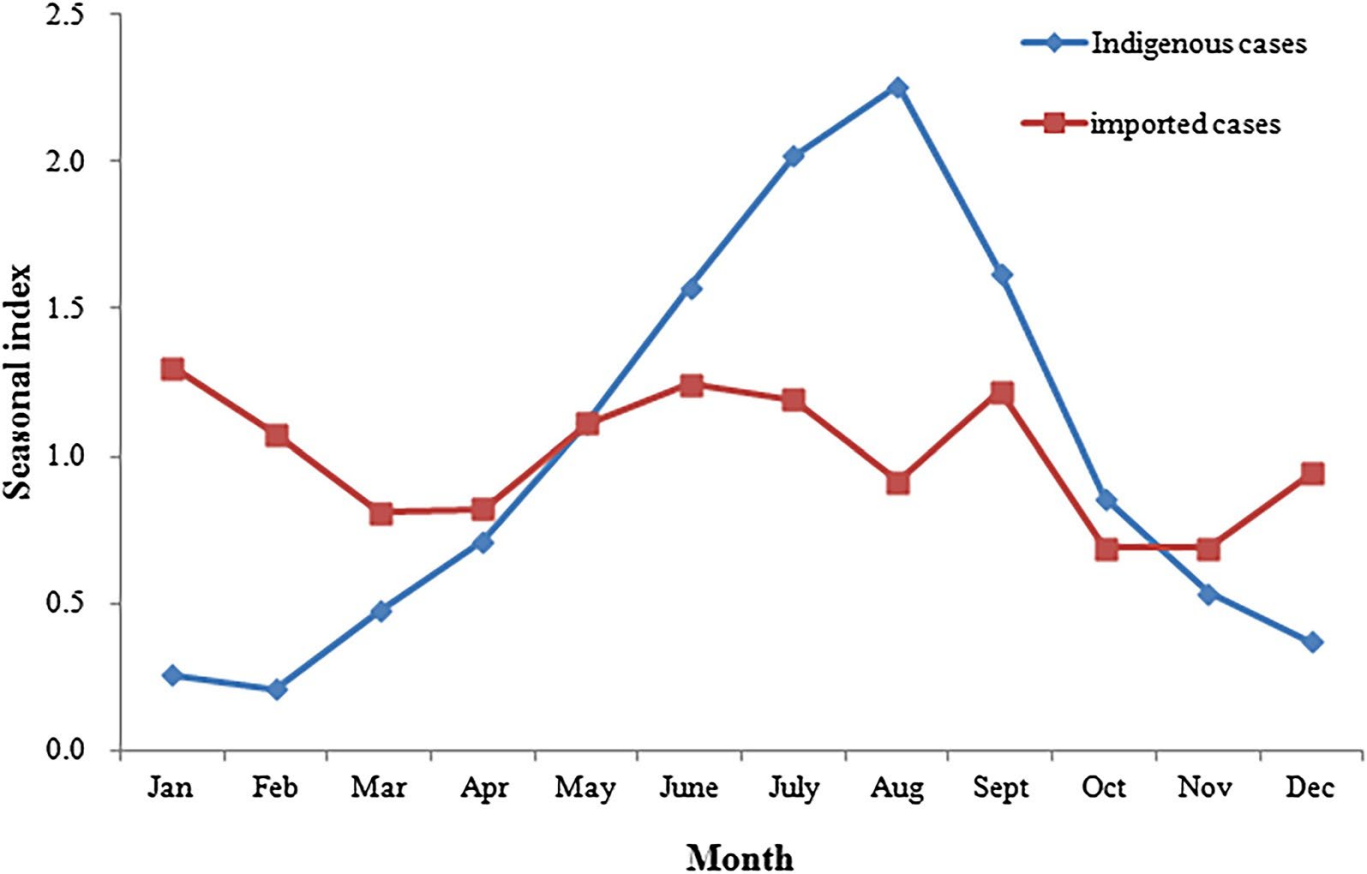
The seasonal index of indigenous malaria and imported malaria in Hubei Province, 2005–2016

Indigenous malaria cases showed a clear seasonal fluctuation during the study period (Fig. 3). A relatively high seasonal index of indigenous malaria was observed from May to September, during which 71.6% (5154/7203) of all indigenous cases were reported. The seasonal index reached a peak in August (2.26). For imported cases, two peaks (one in January to February and a second one in May to September) were observed. There was significant difference in the proportion of monthly cases between indigenous and imported malaria (χ^2^ = 106.28, p < 0.0001).

### Origin of the infection regions of imported malaria cases

Among 906 imported malaria cases from 2005 to 2016 in Hubei, 879 (97.0%) cases had country information available on the origin of infection acquisition. The 879 imported malaria case infections were acquired from 44 countries located in Africa, Asia, Oceania, and South America. 81.1% (713/879) were from Africa during 2005–2016, whereas 18.5% (163/879) originated from Asia; 94.1% (556/591) of *P. falciparum* predominated in imported cases from Africa; Nigeria, Angola, Equatorial Guinea, and Congo (Kinshasa) were the major imported source; 57.2% (123/215) of vivax malaria cases originated from Asia, where Myanmar comprised the majority of imported cases (Table 2).

**Table 2:** Imported malaria country of acquisition in Hubei Province during 2005–2016 by *Plasmodium* species

Countries of origin	*P. falciparum*	%	*P. vivax*	%	Other *Plasmodium*^a^	%	Total	%
Africa	556	94.08	89	41.40	68	93.15	713	81.11
Nigeria	92	15.57	2	0.93	8	10.96	102	11.60
Angola	56	9.48	1	0.47	6	8.22	63	7.17
Equatorial Guinea	46	7.78	7	3.26	6	8.22	59	6.71
Congo (Kinshasa)	46	7.78	3	1.40	7	9.59	56	6.37
Liberia	45	7.61	5	2.33	4	5.48	54	6.14
Ethiopia	4	0.68	44	20.47	2	2.74	50	5.69
Congo (Brazzaville)	31	5.25	3	1.40	10	13.70	44	5.01
Ghana	26	4.40	2	0.93	3	4.11	31	3.53
Guinea	24	4.06	3	1.40	3	4.11	30	3.41
Uganda	21	3.55	2	0.93	4	5.48	27	3.07
Mozambique	23	3.89	0	0.00	2	2.74	25	2.84
Zambia	22	3.72	1	0.47	0	0.00	23	2.62
Cameroon	18	3.05	2	0.93	2	2.74	22	2.50
Other African countries	102	17.26	14	6.51	11	15.07	127	14.45
Asia	35	5.92	123	57.21	5	6.85	163	18.54
Myanmar	19	3.21	74	34.42	3	4.11	96	10.92
Cambodia	5	0.85	21	9.77	0	0.00	26	2.96
Indonesia	7	1.18	9	4.19	0	0.00	16	1.82
Pakistan	0	0.00	13	6.05	0	0.00	13	1.48
Other Asian countries	4	0.68	6	2.79	2	2.74	12	1.37
Oceania	0	0.00	2	0.93	0	0.00	2	0.23
Papua New Guinea	0	0.00	2	0.93	0	0.00	2	0.23
South America	0	0.00	1	0.47	0	0.00	1	0.11
Guyana	0	0.00	1	0.47	0	0.00	1	0.11
Total	591	100.00	215	100.00	73	100.00	879	100.00

^a^Others contained *Plasmodium ovale*, *Plasmodium malariae*, and mixed infection cases

### Spatiotemporal cluster analysis

**Fig. 4:**
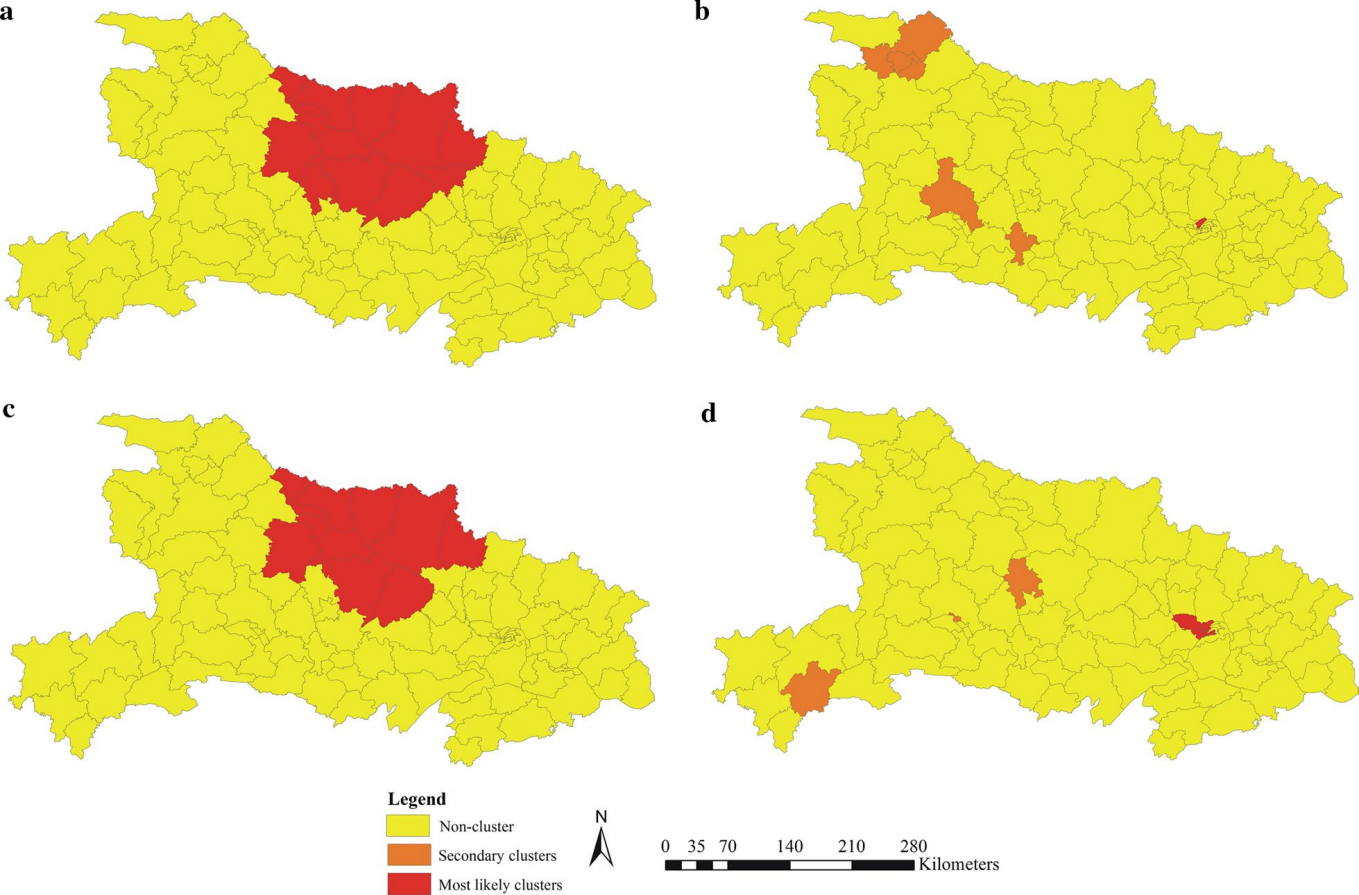
Clusters of malaria cases by indigenous and imported cases in Hubei Province, 2005–2016. **a** Indigenous cases in control stage. **b** Imported cases in control stage. **c** Indigenous cases in elimination stage. **d** Imported cases in elimination stage

The spatiotemporal cluster analysis of malaria cases showed that both indigenous and imported cases were not randomly distributed throughout the study period. During the control stage, the most likely cluster of indigenous cases covered 13 counties. This cluster had 4273 cases with a relative risk (RR) of 18.58 (p < 0.001) and accounted for 58.8% (4273/7270) of the total cases during the control stage. Four different significant spatial clusters were identified for imported cases during the control stage (the most likely cluster and three secondary clusters). The most likely cluster included one county, in Wuhan City. Three secondary clusters covered five counties, which were located in Yichang City (1 county), Shiyan City (3 county), and Jingzhou City (1 county) (Fig. 4, Table 3).

**Table 3:** Clusters of indigenous and imported malaria cases in Hubei Province, 2005–2016

Phase	Infection source	Type of cluster	No. counties	Radius (km)	Time frame	Number of cases	Expected cases	Relative risk	Log likelihood ratio	*P* value
2005–2010	Indigenous	A	13	119.54	2005/4/1 to 2007/11/30	4273	532.72	18.58	6510.03	0.000
	Imported	A	1	0	2007/9/1 to 2007/9/30	24	0.026	1071.73	141.41	0.000
		B_1	1	0	2008/4/1 to 2008/4/30	5	0.022	233.49	22.21	0.000
		B_2	3	38.06	2010/6/1 to 2010/8/31	7	0.12	61.33	21.77	0.000
		B_3	1	0	2008/6/1 to 2010/11/30	11	0.63	18.84	21.52	0.000
2011–2016	Indigenous	A	11	116.4	2011/1/1 to 2011/9/30	68	4.95	54.99	149.05	0.000
	Imported	A	6	16.43	2012/8/1 to 2015/7/31	159	32.66	5.91	137.17	0.000
		B_1	1	0	2012/6/1 to 2015/5/31	40	3.31	12.71	63.90	0.000
		B_2	1	0	2012/3/1 to 2014/4/30	13	1.68	7.83	15.33	0.007
		B_3	1	0	2014/12/1 to 2015/1/31	5	0.12	43.56	13.97	0.022

A: Most likely cluster; B: Secondary cluster

During the elimination stage (2011–2016), the most likely cluster of indigenous cases included 11 counties. For imported cases during the elimination phase, the most significant cluster covered 6 counties, all in Wuhan City; three secondary clusters included three county, in Yichang, Jingmen and Enshi cities (Fig. 4, Table 3).

## Discussion

The study analysed longitudinal surveillance data from 2005 to 2016 to determine changes in the epidemiological characteristics of malaria in Hubei Province during disease control and elimination stages. The study revealed a remarkable decrease in indigenous malaria cases in this region over the past 12 years; in contrast, the number of imported malaria cases increased significantly during the study period.

In the control stage, malaria cases decreased since 2008. A total of 429 malaria cases were reported in 2010, which was the lowest number of malaria cases in control stage. Malaria prevalence was effectively controlled by the high levels of coverage of interventions [5], including mass drug administration, insecticide-treated mosquito net distribution and spraying insecticide indoors [16, 17]. During the elimination stage, no locally transmitted malaria was maintained from 2013 to 2016 with the implementation of NMEP. The strategy of malaria elimination emphasized detection, investigation and disposal of malaria cases and foci, and the ‘1–3–7’ strategy was implemented to ensure effective case detection and confirmation, case classification and treatment, and focus investigation and action [18].

The decrease of malaria prevalence over the past 12 years in Hubei is driven by two factors. One factor was the implementation of the Global Fund to Fight AIDS, Tuberculosis and Malaria, from 2003 to 2012. These initiatives strengthened anti-malaria measures, such as active case detection, standard treatment of malaria patients, improvements in the direct reporting network system of malaria cases, the training of more rural physicians, health education and promotion of malaria in high-risk populations, and vector control in high-transmission areas [19–21]. Another factor was the cooperative malaria control between Hubei and four other provinces (Jiangsu, Shandong, Henan, Anhui) since 1974, which facilitated the application of unified measures, regular exchange of experience and information, as well as annual inspection and monitoring [22].

Imported malaria cases accounted for most cases during the elimination stage, and *P. falciparum* was the predominant species in these cases. The majority of imported cases in Hubei Province originated in African countries with endemic malaria where *P. falciparum* is the main parasite [23–25]. *P. falciparum* infection typically leads to severe disease and death. It has been reported that severe malaria was associated with medical staff not recognizing malaria at an early stage [26–28]. Hubei Province has not had any indigenous cases of *P. falciparum* malaria since 1963, and therefore a lack of awareness of the disease exists in medical staff, which compromises their ability to diagnose and treat these cases. Malaria-related deaths have been reported in Hubei Province [27]. Early discovery, diagnosis and treatment should be prioritized for imported malaria cases [29], and healthcare workers should pay more attention to diagnosis and treatment of imported cases, particularly those originating in Africa.

Imported malaria was more widespread during elimination stage than during control stage. It was identified in four counties in 2005; this increased to 47 counties in 2016. Malaria re-introduction caused by imported cases has been reported in areas that are non-endemic for malaria [30–32]. Imported malaria may lead to local transmission in some areas of Hubei where transmitting vectors remain [33, 34], posing a great challenge to malaria elimination in the province. Enhanced surveillance systems should be sustained to maintain elimination of local transmission in Hubei Province by monitoring malaria importation and potential transmission in risk areas [35]. Moreover, cooperation between public health, health care, immigration and quarantine services, the commercial sector, and police will likely play an important role in the prevention, detection and management of imported malaria [25].

The analysis of this study showed a weak seasonal fluctuation for imported malaria cases (with peaks occurring in January to February and May to September). Chinese New Year holidays are celebrated in January/February, and workers return to China from overseas countries to engage in agricultural work during May to September [36, 37]. Lai et al. reported higher case mortality rates for imported malaria cases in January and February in China [38].

Space–time scan statistical analysis can be used to detected high-risk areas, which provide significant references to prioritize the resource assignment in malaria elimination. The clusters of indigenous malaria cases were seen in 11 counties (Zaoyang, Xiangzhou, Zengdu, Yicheng, Xiangcheng, Fancheng, Zhongxiang, Laohekou, Nanzhang, Guangshui, Jingshan) during control and elimination stages. The two most important vector species for malaria transmission, *Anopheles sinensis* and *Anopheles anthropophagus,* have existed in most parts of these cluster areas for a long time [39], and malaria epidemics have occurred frequently in these areas over past decades [11]. However, a cluster of indigenous malaria cases has not been identified since September 2011.

The most likely cluster of imported cases was located in six counties of Wuhan City (Qiaokou, Jianghan, Hanyang, Jiangan, Wuchang, Dongxihu) from 2005 to 2016. As this region has better medical care than the other cities of Hubei, many overseas labourers return to this area [23, 40]. The secondary clusters were detected in the Yichang, Jingmen, Shiyan, Jingzhou, and Enshi cities. In these cities, many labour service and construction companies offer overseas job opportunities in Africa and Southeast Asia, both of which have endemic malaria [41]. Overseas labourers generally work outdoors at construction sites, they lack awareness of the risks of malaria and personal protection. Interventions strengthening their awareness of the risks of malaria and occupation-based vector control measures should be implemented to reduce the risk of malaria infection among export labourers [42, 43].

There were two limitations to this study. First, under-reporting and misreporting of malaria cases may have existed during the study period, this has been attributed to asymptomatic infection, capacity of malaria testing, and the availability of a healthcare facility. Second, a few imported cases missing information regarding the origin of the infection regions during the control period may have affected the observed origin of imported malaria cases.

## Conclusions

The prevalence of indigenous malaria has declined dramatically over the past 12 years in Hubei Province, China. Although no indigenous malaria cases have been reported in Hubei since 2013, maintaining the status of non-locally transmitted malaria faces some challenges, such as the increase in the number of imported malaria cases and an increasingly widespread distribution of imported cases across the province. Accordingly, the management of imported malaria, prevention strategies for re-introduction of malaria, and malaria surveillance should be strengthened.

## References

[1] WHO (2016). World malaria report 2016.

[2] Yin JH, Zhou SS, Xia ZG, Wang RB, Qian YJ, Yang WZ (2014). Historical patterns of malaria transmission in China. Adv Parasitol.

[3] Sun JL, Zhou S, Geng QB, Zhang Q, Zhang ZK, Zheng CJ (2016). Comparative evaluation of the diagnosis, reporting and investigation of malaria cases in China, 2005–2014: transition from control to elimination for the national malaria programme. Infect Dis Poverty..

[4] Ministry of Health (2010). National malaria elimination action plan (2010–2020).

[5] Cao J, Zhou SS, Zhou HY, Yu XB, Tang HL, Gao Q (2013). Malaria from control to elimination in China: transition of goal, strategy and interventions. Chin J Schisto Control..

[6] Cao J, Sturrock HJ, Cotter C, Zhou S, Zhou H, Liu Y (2014). Communicating and monitoring surveillance and response activities for malaria elimination: China’s “1–3–7” strategy. PLoS Med..

[7] Feng XY, Xia ZG, Vong S, Yang WZ, Zhou SS (2014). Surveillance and response to drive the national malaria elimination program. Adv Parasitol.

[8] Hu T, Liu YB, Zhang SS, Xia ZG, Zhou SS, Yan J (2016). Shrinking the malaria map in China: measuring the progress of the National Malaria Elimination Programme. Infect Dis Poverty..

[9] Lai SJ, Li ZJ, Wardrop NA, Sun JL, Head MG, Huang ZJ (2017). Malaria in China, 2011–2015: an observational study. Bull World Health Organ.

[10] Pei SJ, Huang GQ, Gui AF, Zuo SL, Chen GY, Hu LQ (2004). Analysis of malaria epidemic situation in the past ten years in Hubei Province. J Pub Health Prev Med..

[11] Yuan FY, Huang GQ, Zhang HX, Pei SJ, Liu JY, Hu LQ (2010). Status of malaria epidemic and feasibility of malaria elimination in Hubei. J Pub Health Prev Med..

[12] Zhang Q, Lai SJ, Zheng CJ, Zhang HL, Zhou S, Hu WB (2014). The epidemiology of *Plasmodium vivax* and *Plasmodium falciparum* malaria in China, 2004–2012: from intensified control to elimination. Malar J..

[13] Kong XL, Liu X, Tu H, Xu Y, Jb Niu, Wang YB (2017). Malaria control and prevention towards elimination: data from an eleven-year surveillance in Shandong Province, China. Malar J..

[14] Kulldorff M, Nagarwalla N (1995). Spatial disease clusters: detection and inference. Stat Med.

[15] Kulldorff M (1997). A spatial scan statistic. Commun Stat Theory Methods..

[16] Li KJ, Cai SX, Lin W, Xia J, Pei SJ, Zhang HX (2016). Analysis of malaria epidemic situation and control in Hubei Province from 1974 to 2015. Chin J Schisto Control..

[17] Tang HL (2009). Malaria in China: from control to elimination. Int J Med Parasit Dis..

[18] Yang WZ, Zhou XN (2016). New challenges of malaria elimination in China. Chin J Prev Med..

[19] Pei SJ, Yuan FY, Huang GQ, Gui AF, Zuo SL, Chen GY (2007). Evaluation of results in implementation of Global Fund on Malaria Control in Hubei Province in last three years. China Trop Med.

[20] Gui AF, Huang GQ, Pei SJ, Zuo SL, Chen GY, Hu LQ (2007). Mid-term evaluation on the Hubei global fund of malaria control project. J Pub Health Prev Med..

[21] Wang RB, Zhang QF, Zheng B, Xia ZG, Zhou SS, Tang LH (2014). Transition from control to elimination: impact of the 10-year global fund project on malaria control and elimination in China. Adv Parasitol.

[22] Shang LY, Gao Q, Liu X, Shen YZ, Huang GQ (2006). Evaluation on the effect of cooperative malaria control in 5 provinces of central China in 30 years. J Pathog Biol..

[23] Xia J, Cai SX, Lin W, Pei SJ, Li KJ, Sun LC (2016). Epidemiological analysis of malaria prevalence in Hubei Province from 2010 to 2014. Chin J Schisto Control..

[24] Zhou S, Li ZJ, Cotter C, Zheng CJ, Zhang Q, Li HZ (2016). Trends of imported malaria in China 2010–2014: analysis of surveillance data. Malar J..

[25] Li ZJ, Zhang Q, Zheng CJ, Zhou S, Sun JL, Zhang ZK (2016). Epidemiologic features of overseas imported malaria in the People’s Republic of China. Malar J..

[26] Noor AM, Kinyoki DK, Mundia CW, Kabaria CW, Mutua JW, Alegana VA (2014). The changing risk of *Plasmodium falciparum* malaria infection in Africa: 2000–2010: a spatial and temporal analysis of transmission intensity. Lancet.

[27] Li KJ, Huang GQ, Zhang HX, Lin W, Dong XR, Pi Q (2013). Epidemic situation and control strategy of imported malaria in Hubei Province from 2006 to 2011. Chin J Schisto Control..

[28] Zhang Q, Geng QB, Sun JL, Zhang ZK, Lai SJ, Zhou S (2016). Epidemiological analysis of the deaths of malaria in China, 2005–2014. Chin J Prev Med..

[29] Zhou X, Yap PL, Tanner M, Bergquist R, Utzinger J, Zhou XN (2016). Surveillance and response systems for elimination of tropical diseases: summary of a thematic series in infectious diseases of poverty. Infect Dis Poverty..

[30] Zucker JR (1996). Changing patterns of autochthonous malaria transmission in the United States: a review of recent outbreaks. Emerg Infect Dis.

[31] Lee YC, Tang CS, Ang LW, Han HK, James L, Goh KT (2009). Epidemiological characteristics of imported and locally-acquired malaria in Singapore. Ann Acad Med Singapore.

[32] Danis K, Lenglet A, Tseroni M, Baka A, Tsiodras S, Bonovas S (2013). Malaria in Greece: historical and current reflections on a re-emerging vector borne disease. Travel Med Infect Dis..

[33] Li KJ, Shang XP, Pi Q, Zhang HX, Tong L (2015). Analysis on the density and ecological habit of the main malaria vector Anopheles in Hubei Province. Int J Med Parasit Dis..

[34] Zhang C, Liu JY, Wan L, Li KJ, Lv GY (2015). Epidemic analysis of malaria surveillance sites in Hubei Province from 2012 to 2014. J Hubei Univ Chin Med.

[35] Wesolowski A, Eagle N, Tatem AJ, Smith DL, Noor AM, Snow RW (2012). Quantifying the impact of human mobility on malaria. Science.

[36] Clements AC, Barnett AG, Cheng ZW, Snow RW, Zhou HN (2009). Space-time variation of malaria incidence in Yunnan Province, China. Malar J..

[37] Wardrop NA, Barnett AG, Atkinson JA, Clements AC (2013). *Plasmodium vivax* malaria incidence over time and its association with temperature and rainfall in four counties of Yunnan Province, China. Malar J..

[38] Lai SJ, Wardrop NA, Huang ZJ, Bosco C, Sun JL, Bird T (2016). *Plasmodium falciparum* malaria importation from Africa to China and its mortality: an analysis of driving factors. Sci Rep..

[39] Xia J, Cai SX, Zhang HX, Lin W, Fan YZ, Qiu J (2015). Spatial, temporal, and spatiotemporal analysis of malaria in Hubei Province, China from 2004–2011. Malar J..

[40] Wu K, Yang Y, Zhou SM, Xu MX (2015). Epidemiological analysis of overseas imported malaria in Wuhan City. Chin J Schisto Control..

[41] Huang GQ, Li KJ, Zhang HX, Yang Y, Wu K, Cao YJ (2014). Investigation and analysis of malaria prevention and control measures for migrant workers to high prevalence area in Hubei province. J Pub Health Prev Med..

[42] Cotter C, Sturrock HJ, Hsiang MS, Liu J, Phillips AA, Hwang J (2013). The changing epidemiology of malaria elimination: new strategies for new challenges. Lancet.

[43] Moonen B, Cohen JM, Snow RW, Slutsker L, Drakeley C, Smith DL (2010). Operational strategies to achieve and maintain malaria elimination. Lancet.

